# The Complexities of Managing Gestational Diabetes in Women of Culturally and Linguistically Diverse Backgrounds: A Qualitative Study of Women’s Experiences

**DOI:** 10.3390/nu15041053

**Published:** 2023-02-20

**Authors:** Melissa Oxlad, Sharni Whitburn, Jessica A. Grieger

**Affiliations:** 1 School of Psychology, The University of Adelaide, Adelaide, SA 5000, Australia; 2 Adelaide Medical School, The University of Adelaide, Adelaide, SA 5000, Australia; 3 Robinson Research Institute, The University of Adelaide, Adelaide, SA 5005, Australia

**Keywords:** gestational diabetes, culturally and linguistically diverse, lifestyle management, dietary advice, education, women, qualitative

## Abstract

Aim: This study aimed to explore women’s perspectives and experiences concerning how culture impacts the lifestyle management of gestational diabetes mellitus (GDM) in women of culturally and linguistically diverse (CALD) backgrounds. Methods: Women of any cultural background diagnosed with GDM within the previous 12 months were purposively recruited from two Australian metropolitan hospitals. Data collected using semi-structured interviews (*n* = 18) and focus groups (*n* = 15 women in three groups) were analysed using reflexive thematic analysis. Results: Three themes were generated: “cultural beliefs and obligations impact lifestyle management of gestational diabetes”, which describes how some cultures lack awareness about GDM, and modifications or restrictions were viewed as depriving the infant, but sometimes adaptions could be made so that a culturally appropriate meal was suitable for GDM management; “the relationship between cultural foods and gestational diabetes management”, which discusses how important cultural foods may be incompatible with appropriate GDM management, so women worked to find solutions; “gestational diabetes education lacks cultural awareness and sensitivity”, which illustrates how current education fails to address differences in cultural beliefs, language and eating practices. Conclusion: Cultural beliefs, obligations and food practices must be considered when assisting women of CALD backgrounds using lifestyle modification to manage GDM. GDM education must be culturally sensitive and competent and, where possible, be delivered by health professionals of a shared cultural group.

## 1. Introduction

Gestational diabetes mellitus (GDM) is the onset or first recognition of glucose intolerance during pregnancy, mostly in the second or third trimester [1]. The prevalence of GDM in Australia has tripled over the last 15 years and has now reached 15% [2]. Women with GDM are at a 10 times higher risk of developing type 2 diabetes 5–10 years post-partum [3] and are 2–3 times more likely to develop ischemic heart disease and hypertension up to 25 years after birth [4]. In addition, the offspring of women with GDM are more likely to be born large for gestational age, have a higher body mass index in childhood [5] and be more insulin resistant in adolescence [6].

Current first-line clinical management of GDM is a healthy diet and exercise; however, there are no universal guidelines for its management [7]. Although diabetes educators and dietitians demonstrate consistent knowledge of nutritional management for GDM and uniform delivery methods, concerns exist that women with GDM struggle to follow nutritional advice and often disengage from services [8]. In a systematic review of 10 studies, He et al. [9] noted that women with GDM had difficulty in changing their eating habits because the recommended eating plan differed significantly from their previous dietary habits. In a review among Black, Asian and minority ethnic populations, the nutritional education provided was seen to contradict traditional beliefs about pregnancy, contributing to anxiety, psychological distress and potentially exacerbating underlying health fears associated with race [10]. These findings highlight the disconnection between the delivery of recommendations for GDM by health professionals and the uptake of nutritional advice by women who have GDM.

Culture offers a potential explanation for some of this difficulty, with a person’s diet being culturally situated. Each culture has norms about foods and eating practices. Adhering to a prescribed nutrition regimen may be more difficult for women from minority ethnic groups than for women from the ethnic majority. In 2019, in Australia, 4.8% of women were Aboriginal or Torres Strait Islander, and 36% of women who gave birth were not born in Australia [11]. Such diversity brings different expectations, knowledge and practices about antenatal care. Immigrant women in Australia reported that the dietary advice they received about GDM was challenging [12], there was difficulty in meeting the demands to change the diet [13], and the dietary advice lacked cultural relevance [14]. Yet, other cultural groups identified GDM as an opportunity to improve their health [15]. In some cultures, GDM is unheard of or goes unspoken. Therefore, when diagnosed, women experience a sense of isolation [16], are often left to do their own research and may seek advice within their communities [16,17], or the advice they receive conflicts with their cultural practices and beliefs regarding pregnancy [12].

There is a growing body of literature concerning White and culturally and linguistically diverse (CALD) women’s GDM experiences in Australia, but it is currently insufficient to offer effective culturally responsive communication and care. Furthermore, there is far less information on how culture impacts the lifestyle management of GDM in women of CALD backgrounds in Australia. Therefore, barriers to dietary adherence and the influence of cultural diversity need to be identified to support implementation, aid practical resources and increase dietary adherence. Thus, we aimed to explore women’s perspectives and experiences concerning how culture impacts the lifestyle management of GDM in women of CALD backgrounds.

## 2. Materials and Methods

### 2.1. Design, Setting and Sample Recruitment

We employed a qualitative design using focus groups and semi-structured interviews. Participants were recruited using purposeful selection from two large metropolitan hospitals in Adelaide, Australia. A research nurse employed at each hospital identified potentially eligible women from hospital records; women required a GDM diagnosis within the past 12 months and the ability to speak and understand English. The research nurse telephoned potential participants to describe the study, and interested women gave verbal consent to be sent an information sheet and consent form. We used face-to-face focus groups and semi-structured telephone interviews as many women could not attend focus groups due to lack of transport or their husbands’ preference. Written consent was obtained before the commencement of the focus groups and was verbally recorded at the start of individual interviews. Interviews and focus groups were conducted between November 2021 and June 2022. The University of Adelaide Human Research Ethics Committee approved this study (2021/HRE00128).

Focus groups and interviews were guided by an open-ended question interview guide informed by previous literature studies. The topics covered included demographic information and women’s perspectives about managing GDM. Example questions included ”Can you tell us briefly about how you managed or aimed to manage your GDM?”, ”What were the greatest difficulties or barriers that you encountered when diagnosed with GDM?“ and ”Can you please describe your experience with the food guidance that you were provided following the diagnosis of GDM? Were there any issues in relation to your culture/food preference or food availability that made following these guidelines difficult?”

Focus groups were facilitated by a research fellow with a nutrition background, while a paediatric diabetes nurse educator or a midwife conducted the interviews. With the participants’ consent, focus groups and interviews were audio-recorded and transcribed verbatim, with identifying information being removed from transcripts. Participants received AUD 50 gift cards as a sign of gratitude for their involvement.

In line with best-practice qualitative research involving ”member reflections” [18], all women were offered the opportunity to review their focus group/interview transcript and/or a summary of the themes; none chose to do so. We did not seek data saturation, as this concept does not align with the values and assumptions of reflexive thematic analysis [19]. Rather, Braun and Clarke [19] recommend that data collection continues until sufficient meaning can be generated. All authors felt that this was achieved in our sample.

In line with best-practice qualitative research, we recognised the researchers’ potential to influence the research process, including data collection, analysis and interpretation, so we engaged in self-reflexivity throughout the research project [18,20]. We are a team of women researchers with experience in conducting reproductive health-related research. The first and third authors are mothers; the first author has experience with GDM.

### 2.2. Data Analysis

Data were analysed using six-step reflexive thematic analysis [20]. That is, the analysis involved (1) data familiarization by reading the transcripts multiple times; (2) generating initial codes relevant to the research aims; (3) collating related codes to generate potential themes; (4) reviewing potential themes in relation to the research aims and ensuring they accurately reflected the data; (5) refining and naming themes; and (6) selecting extracts to illustrate the themes and writing up the results. Given the study’s exploratory nature, we employed an inductive approach. The first and second authors independently familiarized themselves with the data and undertook initial coding. After discussing coding, the first author developed the initial themes, which all authors then reviewed and refined. All authors agreed on the final themes.

## 3. Results

### 3.1. Participants

The participants were 33 post-partum women (18 individual interviewees and 15 focus group participants) aged 20–42 years (*M* = 32.09, *SD* = 4.67). Focus groups ran for 59–71 min (*M* = 65.33 min), while interviews ranged from 10 to 28 min (*M* = 14.39 min). Women represented multiple ethnicities: Indian (*n* = 11), Australian (*n* = 6), Caucasian (*n* = 4), English (*n* = 2), Pakistani (*n* = 2) Aboriginal (*n* = 1), Bangladeshi (*n* = 1), Colombian (*n* = 1), Filipino/Italian (*n* = 1), Filipino/Spanish (*n* = 1), Iranian (*n* = 1), Iraqi (*n* = 1) and Nepalese (*n* = 1). Parity ranged between primiparous and four; three women had had GDM in two pregnancies.

### 3.2. Themes

We generated three themes that demonstrate how culture adds complexity to the lifestyle management of GDM.

#### 3.2.1. Cultural Beliefs and Obligations Impact Lifestyle Management of Gestational Diabetes

Women were very motivated to follow lifestyle advice for their health and that of their baby; however, culture was an important influence on efforts to manage GDM with lifestyle change. Women described how cultural knowledge, beliefs and obligations were integral to understanding and managing GDM. They noted that GDM is not something people in all cultures are aware of; further, rates of GDM in some cultures were low, meaning that women often had to educate themselves and their families about the condition. In some cases, women also questioned whether countries have different approaches to pregnancy care.

*Mostly in our culture people don’t have diabetes—gestational diabetes. I don’t know why, but in my family I’m the first one who have gestational diabetes. Even my mum said we didn’t hear about gestational diabetes. I told her that maybe we don’t do in Pakistan the proper treatment, we don’t do the test and everything…I think here we go on a proper way in Australia, to do all the process and tests…Maybe in Pakistan they don’t worry about these things.*

Additionally, cultural views about the importance of food in general and the role of food in pregnancy and infant health were also expressed. For example, women described how family members valued eating during pregnancy and questioned the need to restrict foods, seeing eating as beneficial to the child and pregnancy as a time to eat freely.

*Because my parents are Polish, so they’re also from a background of eat anything, you’re meant to be eating because you’ve got a child, you shouldn’t have to worry about that.*

In addition, when women tried to modify their diet to manage their GDM, this was not always met with understanding. Instead, family members who lacked awareness of or who had not been screened for GDM during their pregnancies did not see a need for lifestyle changes and encouraged women to continue eating whatever they wanted.

*Like I tell my mum, I’ve been diagnosed. She’s like, ‘we didn’t have any of this in the ‘80s. We just ate whatever. Just eat whatever’. I have to say, ‘I’m avoiding salami. I’m avoiding soft cheese’. She’s like, ‘what are you doing? Just eat whatever. We never had these tests.’ So then that’s sort of playing in my mind. Like how do you get out of that when they have no idea but they just had a baby and they weren’t told about anything to do with diabetes.*

Additionally, any dietary modifications or restrictions were viewed as depriving the infant. Women articulated that they needed to educate family members about the need for lifestyle changes.

*So my partner’s from cultural—he’s Indian background, so there’s a big belief in don’t cut anything out, keep eating the way you are, eat, eat, eat, sugar, sugar, sugar, or it’s very vegetarian based…Yeah, so culturally, they don’t understand it in a way. So it’s, you’re taking something away from the baby, or blah, blah, blah, and I’d have to try and explain to them, no, I’m not. In a way, it’s just a better lifestyle, a heathier lifestyle.*

In making necessary dietary changes, women also spoke of how this impacted family eating practices, with meal preparation often being framed as challenging. In some instances, women could make adaptations, so a culturally appropriate meal could also be suitable for GDM management. For example, avoiding breads or using homemade breads, substitutions or eating smaller portions.

*We must have a curry—curry with bread or curry with rice, something like that. So I just make our own bread for myself. I just went out and make the bread for curry—and something like a soup, chicken soup and tomato soup, something like that.*

In other instances, women prepared their own meal or needed to make one meal for themselves and a separate meal for other family members—this arose due to food preferences or cultural expectations about supplying a culturally appropriate meal.

*I had to make myself a separate meal with more veggies because my children are not really—they didn’t like what I was eating, it was all boiled. So, I had to make myself a separate meal.*

In contrast, some women prepared the meal best suited to them for their immediate family. However, one woman noted that while her husband was supportive, this approach would not have been acceptable if family were visiting.

*What I eat gets cooked and everybody else in the family gets to eat that…not that my husband’s complaining, he loves the fact that we’re not having any Indian food…if I had my family over then it would have been a challenge because then we’d have to cook two meals, different for me and different for the elders of the family.*

Culture and its influence on diet, eating practices and family obligations is an important consideration in the lifestyle management of GDM in women from CALD backgrounds. However, while culture governs attitudes towards food and eating practices in pregnancy, culturally important foods also have a significant role in the lifestyle management of GDM.

#### 3.2.2. The Relationship between Cultural Foods and Gestational Diabetes Management

Women described how important cultural foods may be incompatible with appropriate GDM management and how they worked to find solutions. Many important and frequently eaten foods were incompatible with optimal management. For example, one woman of Indian culture noted that in *“a lot of food that we eat at home, and not just Indian culture, but a lot of traditional cultures of food is very, very carb-rich and carb-dense”*, and that for her culture, *“the food that we eat, the Indian food, it just doesn’t work with GDM”*. In Indian culture, while many people are vegetarian, there is also a heavy reliance on flatbreads (i.e., roti and chapati) containing sugar and high-carbohydrate white rice, lentils and dahl. Women described receiving varying information about consuming cultural foods, with some women reporting being advised to avoid certain cultural foods.

*The chart was basically not Indian food…There were two Indian ladies, we both asked what should we eat as breakfast and then dinner. Basically, we have to avoid all bread, like the roti, that is the main thing we have to avoid.*

In contrast, other women reported receiving advice that some cultural foods could still be eaten after consideration of portion size and timing. For example, “*the intake and the time and how much we take that’s the main thing”*. Women found creative ways to navigate potential incompatibilities between cultural foods and GDM management, including preparing meals in a way that is *“respecting the culture”*; cooking differently (i.e., more stir-frying, boiling and grilling), *“so, the cooking—the preparation and the method was really different”*; substituting foods (i.e., multi-grain flour instead of wheat flour, sweet potatoes instead of potatoes, and brown rice instead of white rice); eating small amounts more frequently; and reducing quantities and portion sizes.

*Probably being Indian I would say our cultural food involves a lot of flatbread which is high on sugar. So, I would probably just say that cut down on the flatbread, eat more veggies…Instead, for example, if you take three flatbreads with the veggies, cut down to one, eat more veggies.*

The strategy of reducing quantities and portion sizes was also used as a means to minimize dietary changes and hold on to pre-diagnosis eating practices.

*I was eating the same food that I was eating earlier...I just reduced the quantities. If I’m taking rice three cups, then I’m just taking rice one cup and instead of that I’m substituting it for vegetables or meat or whatever that I can eat without increasing the sugar levels.*

While many women used food substitutions and reduced consumption to manage their GDM, others significantly changed their food practices. For instance, some vegetarians chose to add meat to reduce carbohydrate intake.

*I had to pretty much chuck vegetarian out the door, because it had a lot of lentils and rice and, unfortunately, it’s a lot of carbohydrates in it. So I went more of a protein-based.*

However, the pull of culture can be very strong, with some women retaining cultural eating practices, viewing these as important for their child, even if inconsistent with scientific evidence.

*There are lots of people who still stick to culture…lots of butter and things like that…people don’t want to change their culture.*

Specific foods and ways of preparing food are highly valued aspects of culture. While women are highly motivated to make lifestyle modifications to manage their GDM, health professionals must recognize that important cultural foods may be incompatible with GDM management. For the most part, women from CALD backgrounds are willing to make changes regarding their cultural foods. However, the need for and challenge in making these changes should be recognised in culturally competent GDM education.

#### 3.2.3. Gestational Diabetes Education Lacks Cultural Awareness and Sensitivity

Women described how GDM education did not address differences in cultural beliefs, language and eating practices; rather, women were educated on foods they could not eat, were unsure how recommendations would fit with their cultural eating practices, noting *“I think from our culture it’s really difficult for us to just go on that one [recommended diet]…because our food is a lot more different from Australian”*, and were often provided with limited or inappropriate alternatives.

*When they were saying ’you won’t be able to have white rice’, the Indian ladies were like, ’oh, what am I going to have instead?’ You could see them just going, ‘well, that’s the base of our meals.’*

Such an approach made it particularly difficult for women whose staple foods consisted of white rice and flatbread, since these are foods to avoid when managing GDM.

*See I love Jasmine rice, we eat a lot of rice with every meal. So the dietitian said to go on Basmati rice. I don’t like Basmati rice.*

The education sessions were only offered in English, which sometimes made it challenging for women when English was not their first language. One woman reflected, “*when you think it’s hard for us, imagine that being your second language… a lot of people who experience GDM are people from culturally and linguistically diverse backgrounds. So, it’s so important to tailor for them”.* Encouragingly, women of CALD backgrounds described how the support from health professionals allowed them to overcome language barriers.

*Yes, so I just want to say that the nutritionists’ behaviour and the way they talked to us, especially when we are from other backgrounds. We don’t have any good English so it’s hard to understand what they tell us or what we are telling them, so it’s too hard. But the way that they managed these difficulties, and they’re always good to us, nice, very kind, it was very helpful, and I hope they continue.*

Women often highlighted the importance of individualized education not just in terms of language but in terms of diet and food practices. Women recognized that even within the same culture, regional differences exist. For example, one woman explained, *“I’m from the northern part of India, salt is different in the east, we are different—every state is different”*, while another stated, *“I’m from India and specifically I’m from the southern part of India. So, if it is from northern part of India they will eat more of wheat, but we normally eat more of the rice.”* Women expressed that such differences should be considered in education.

*You might be part of the same cultural groups and organizations, but you still might eat actually different, so you need that different sort of advice.*

Some women described attending education sessions alongside women who may have a different diet to themselves as daunting and suggested one-on-one support as a valuable alternative.

*I went through the Aboriginal Birthing Program and they were a great support. I’m not too sure if that was something they could also pick up? Because it can be a bit daunting…there’s going to be other people there….a one-on-one for Aboriginal people would be better.*

In addition to one-on-one support, women suggested that it would be useful to have someone to relate to in the education sessions who understood their challenges. Women described this person as an advocate or “*someone who has had GDM before, from a different background*”. Women also emphasized the importance of including their partner in education sessions to assist couples in implementing the suggested recommendations.

*Maybe bring a support person, there’s a lot of information. That sort of thing would’ve been good because I think—particularly if you’re in your own home, like having someone who’s involved in the cooking know what’s going on, what’s the dos and don’ts, would’ve been hugely helpful.*

The clear benefits of having a partner who is willing to learn and also implement changes was illustrated by one woman.

*Yeah, like he [husband] came along to all my appointments with me and took the time to understand what this diagnosis really meant, because we didn’t have any clue about this gestational diabetes as we do not have any family member had this diabetes before. So we were really unsure about what these things are, so he was really interested in understanding this diagnosis…he really did these changes in our daily lifestyle as well, because not only for me, he also changes his lifestyle, food journey, so it was really helpful, because eating alone for different food is really difficult.*

Women highlighted the importance of individualized education that addresses potential language barriers, and cultural beliefs and practices surrounding food. Partners and support people being included in education was also valued.

## 4. Discussion

This study aimed to explore women’s perspectives and experiences concerning how culture impacts the lifestyle management of GDM in women of CALD backgrounds. Women reported that testing and treatment for GDM were low in some countries, resulting in limited understanding and awareness. Additionally, cultural beliefs showed that families value eating during pregnancy, with diet modifications perceived as depriving the baby. This finding concurs with a recent study in South Asian immigrant women in Australia, where women were shocked when advised to decrease their food intake, as this goes against norms such as gaining weight and eating for two [12]. In contrast, a prior study in women who were currently or previously diagnosed with GDM found that when women visit family and friends, they feel obliged to eat the food prepared for them [21] which can potentially compromise their GDM management if the food is of poor diet quality. Some women in our study made a GDM-appropriate meal for themselves and a separate meal for family members to meet cultural obligations. While this may enable GDM management, it may still pose cultural issues and creates extra work for women.

Consistent with previous studies, revealing that it is difficult for women to implement dietary changes when staple cultural foods are ”carbohydrate heavy” [16,21], the women in our study described how common cultural foods were incompatible with their GDM management. Nutritional advice varied, with some women having been advised to avoid these cultural foods completely, while others were informed to eat their cultural foods in smaller portions or at certain times. Conflicting advice such as this occurs when dietitians lack education about variations in cultural cuisines, resulting in women sacrificing these dishes [17,21]. As a result, women undertook research and sought advice within their communities to make substitutions that better replicate their usual diet [16,17]. We observed this solution focus in our sample, where women substituted foods (multi-grain flour instead of wheat flour) and employed alternative cooking methods (boiling and grilling) to enable them to continue eating cultural foods while managing their GDM.

While women identified solutions, the education provided focused on foods to avoid and offered limited alternatives or substitutions and was reported to be only offered in English. Some past literature studies have found that language barriers sometimes lead women to misunderstand information, resulting in problems in following dietary advice and monitoring blood glucose levels [22,23]. However, in our study and a study in local and immigrant women in Norway [23], women with an immigrant background did not report challenges in understanding and following advice. Despite this, health professionals have noted challenges when educating women of CALD backgrounds, attributed to language, culture and religion [12] or a lack of resources for CALD women [8]. Women in our study recommended one-on-one individualized education and advice that was culturally sensitive. They also highlighted that having a support person attend education sessions may increase the support person’s understanding and the likelihood of support in lifestyle changes. This is in line with previous studies that have described the need to include partners, emphasizing difficulties for women in implementing lifestyle changes when their partner is unwilling to participate in any changes [22,24]. We build on these studies, with women in our study expressing a desire to include an advocate or someone who has experienced GDM from their culture.

Culture itself does not appear to influence the ability to benefit from GDM education and manage GDM with lifestyle modifications. Several studies show that women of different ethnicities can effectively manage GDM, for example, Chinese women with GDM living in China [25] and Thai women living in Thailand [26], or where the population was specifically recruited, such as African born [27] or Aboriginal born [28], to understand the experiences and perceptions of a largely homogenous group of women with GDM. In such study, the health professional who provided GDM education was of the same ethnicity as the women participating. Contrastingly, in Australia, GDM education for women of CALD backgrounds is typically provided by White women. This cultural incongruence cannot be overcome through interpreters, as language alone is not the only barrier to suitable education; health professionals also require cultural knowledge and understanding to provide culturally sensitive and competent care. Therefore, it is essential to have health professionals of diverse cultural backgrounds providing GDM education, and in their absence, for White health professionals to be culturally competent.

This study has important implications for policy and practice. In Australia and internationally, the prevalence of GDM is rising. The increased prevalence of GDM poses an increasing public health burden, given the intergenerational cycle that GDM perpetuates [29]. Additionally, in Australia, the population is increasingly comprising more women of reproductive age who are of ethnic minority backgrounds [30]. We have recently highlighted the lack of cultural resources for nutritional management of GDM in women of ethnic minority backgrounds [8], which is consistent with other studies [12,31,32] and demonstrates the ongoing need to consult women of CALD backgrounds about their views, experiences and needs, so that appropriate, evidence-informed health education prevention and management resources, as well as culturally sensitive and appropriate interventions and support, can be offered. Co-developing nutritional management plans that are tailored to the cultural context and that are emphasized by trained diabetes educators and dietitians would enhance current GDM care for women of CALD backgrounds. Recommendations also include training staff to be mindful and sensitive to culture and to consider the importance of cultural obligations and food practices in women’s lives and how such cultural factors impact women’s abilities to manage their GDM. Such training may enable staff to provide quality nutrition education and GDM management strategies that are considerate of and, when supported by medical evidence, compatible with cultural beliefs and practices. In turn, women of CALD backgrounds with GDM who receive education and guidance from culturally competent staff are likely to follow care recommendations and to be more satisfied with their care. Finally, to further enhance the likelihood of optimal GDM management in women of CALD backgrounds, partner and/or family engagement is also recommended; increasing family members’ knowledge about GDM and the changes needed to optimise the health of women and their babies is likely to improve the sustainability and success of well-integrated, culturally appropriate nutrition.

A strength of our study is the cultural diversity of our sample, providing important information on women’s experiences of how culture impacts awareness of and lifestyle management in GDM. The use of focus groups and interviews conducted in multiple locations somewhat mitigated selection bias. This approach also provided the capacity to access marginalized groups who may otherwise be unable to participate using traditional research methods and to gather in-depth accounts and views. Despite the strengths of our study, it is not without limitations. For example, although we recruited women with suitable English proficiency across two major public hospitals in Adelaide, Australia, our findings may not represent the views and experiences of all women diagnosed with GDM in Australia. The need for a degree of English proficiency may have excluded some potential participants and conducting the research in English (not all participants’ first language) may have hampered some participants’ ability to fully articulate their experiences, thereby leaving us with an incomplete understanding of the complexities of managing GDM in women of CALD backgrounds. Although challenging, where possible, it may be beneficial to offer participation in women’s first language. In addition, all women in our study were post-partum and may reflect differently on their experiences than women interviewed when newly diagnosed or actively managing a pregnancy with GDM. Finally, all women were from one Australian metropolitan area, and although women of several ethnicities were included, we cannot know whether women of ethnicities that were not included may have shared similar or disparate views and experiences regarding management of GDM. Therefore, as our findings may not be generalizable to all women of CALD backgrounds in Australia, we recommend that future research seeks the views of women of diverse sociocultural backgrounds, including women of under-represented cultural groups, varying socio-economic and education levels, residing in other Australian cities and rural communities where access to GDM support may vary and at varying stages of pregnancy. And if possible, that such research offers women the opportunity to participate in their first language. This could enable researchers and clinicians to generate a more complete understanding of women’s experiences and to develop evidence-informed resources and support.

## 5. Conclusions

Managing GDM with lifestyle modification can be effective, but culture must be considered, as it adds complexity. Health professionals need to recognise cultural beliefs, obligations, language and cultural eating practices. GDM education must be culturally sensitive and competent and, where possible, be delivered by health professionals of a shared cultural group. It is also essential to include women’s partners or families in education to enhance the understanding of why lifestyle modifications are needed and to increase the likelihood of them being effectively implemented.

## Data Availability

Data are not available due to ethical restrictions.
